# Parental health literacy in anorectal malformation: needs and challenges

**DOI:** 10.1007/s00383-025-06096-6

**Published:** 2025-07-16

**Authors:** Signe Olsbø, Marie Hamilton Larsen, Sara George Kiserud, Trine Sæther Hagen, Åsmund Hermansen, Kristin Bjørnland

**Affiliations:** 1 Department of Gastrointestinal and Pediatric Surgery, Oslo University Hospital, Rikshospitalet, Postboks 4950 Nydalen, 0424 Oslo, Norway; 2 Institute of Clinical Medicine, University of Oslo, Oslo, Norway; 3 Department of Behavioral Medicine, Institute of Basic Medical Science, Faculty of Medicine, University of Oslo, Oslo, Norway; 4 Lovisenberg Diaconal University College, Oslo, Norway; 5 Department of Social Work, Child Welfare and Social Policy, Faculty of Social Sciences, OsloMet—Oslo Metropolitan University, Oslo, Norway

**Keywords:** Anorectal malformation, Health literacy, Parents, Self-efficacy, e-health

## Abstract

**Aim:**

Explore health literacy (HL) among parents of children with anorectal malformation (ARM) and identify the predictors of HL.

**Method:**

Parents of children < 16 years treated for ARM were invited to complete the Health Literacy Questionnaire-Parent (HLQ-p), General Self-efficacy Scale (GSES), electronic Health Literacy-Scale (eHEALS) and a study-specific questionnaire. Demographic data were collected. Ethical approval was obtained.

**Results:**

137 parents (40% fathers) of 105 children (median age 7.1 years) participated. The highest HL scores were in managing the child’s health and engaging with healthcare providers, while scores were lowest in social support, information sufficiency, and interpreting health information. Higher HL correlated with increasing parental age and education. Parents not speaking the native language at home or not living with the child’s other parent had lower HL scores. More challenges were observed among parents of female children and children with comorbidities. Parents had high eHEALS scores (mean 3.7, SD 0.6, max score 5), while 48% had low self-efficacy scores (max score 4).

**Conclusion:**

Many parents experience a lack of information, insufficient social support, and difficulty interpreting information. Predictors of HL challenges include having a female child, a child with comorbidity, younger parental age, lower education, and low self-efficacy. These parents will likely benefit from targeted support.

**Supplementary Information:**

The online version contains supplementary material available at 10.1007/s00383-025-06096-6.

## Introduction

Approximately, 1 in 5000 newborns are born with an anorectal malformation (ARM) [1]. ARM encompasses a spectrum of anomalies of the anus and rectum, ranging from minor to complex deformities. The majority of children with ARM require surgical intervention. The goal of surgery is to correct the anatomical defect and create an anal opening within the anal sphincter complex [1]. Many patients have impaired bowel function after surgery. Up to 80% experience constipation and up to 48% have fecal incontinence [2, 3]. These bowel problems can affect both physical and psychosocial well-being [4].

Managing bowel problems may require several interventions including medications, dietary modifications, bowel-emptying routines, or regular enemas. Often, frequent contacts with healthcare providers (HCP) are necessary as many patients need individually tailored treatment. Since over 75% of ARM patients have associated anomalies, many parents must interact with various HCP and coordinate the child’s healthcare. They must participate in decisions impacting their child's health and well-being. These responsibilities may lead to physical, mental, and social stress, and ultimately affect the family dynamic [5, 6]. Given this context, parental health literacy (HL) is crucial.

The World Health Organization defines HL as the ability to acquire, comprehend, assess, remember, and apply health-related information [7]. Low HL in adults is linked to several adverse outcomes, such as increased hospitalization, poorer disease management, and higher mortality [8]. Parental HL involves efficiently using health information to manage and support the child’s health needs [9]. Parents with higher HL are better equipped to adhere to treatment plans, take part in shared decision-making, effectively use digital health tools, and navigate the healthcare system [10]. Emerging evidence indicates a strong correlation between parental HL and positive health outcomes in children [11]. To our knowledge, HL in parents of children with ARM has not previously been studied. Therefore, the aim of this study was to explore parental HL within the context of ARM. Additionally, we aimed to investigate the demographic factors that may predict HL challenges and examine how child-specific variables and parental self-efficacy influence HL.

## Methods

### Study design and recruitment

We conducted a cross-sectional study, inviting parents of children with ARM under 16 years of age to participate. All patients were treated at Oslo University Hospital, a tertiary referral center treating 10–15 new ARM patients yearly. ARM patients born between 2008 and 2024 were identified through electronic search and through theater logbooks. Primary caregivers were invited either via mailed letters or during outpatient clinic visits from October 2023 to November 2024. Participants had to complete the questionnaires in Norwegian, either online or on paper. A follow-up reminder was sent to non-responders after 3 weeks.

### Measures

#### Patient and parent characteristics

Clinical data for the children of responders were gathered from medical records. This information included details such as type of ARM, comorbidities, and operations. Sociodemographic information about caregivers including educational background and living arrangements was collected through the study-specific questionnaire.

#### Questionnaire on knowledge about anorectal malformations

Eight statements about ARM were included in a self-made, study-specific questionnaire to assess general knowledge about ARM. Four questions were reserved for parents of children over 4 years because certain information becomes relevant as the children get older. Parents rated their agreement with the statements using a 5-point Likert scale. For the analysis, responses of “strongly agree” and “agree” were grouped together as “agree,” while “strongly disagree” and “disagree” were combined as “disagree.”

#### The health literacy questionnaire-parent (HLQ-p)

The HLQ is a generic, multidimensional instrument designed to assess an individual’s HL skills and abilities [12]. We used the parent version of the HLQ (HLQ-p), a validated tool used to evaluate HL levels among parents in relation to the healthcare of their children [9]. The HLQ-p consists of nine domains (Table 2). Responses for domains 1–5 are measured on a 4-point Likert scale ranging from “strongly disagree” to “strongly agree,” while domains 6–9 use a 5-point Likert scale assessing capability/difficulty ranging from “can’t do/always difficult” to “always easy”. We defined having a HL challenge as a score < 2 for domains 1–5 and < 3 for domains 6–9. A total score is not calculated for the HLQ-p scales. Instead, mean scale scores are interpreted separately [12]. The HLQ-p has been validated in Norwegian with satisfactory results [9]. The reliability of the HLQ-p was assessed using Cronbach’s alpha (Cronbach’s α 0.72–0.84).

#### The electronic health literacy scale (eHEALS)

The eHEALS assessed the participants' perceived level of electronic HL (eHL), regarding finding, evaluating, and using online health information [13]. The eight-question survey uses a 5-point Likert scale, with higher scores suggesting a higher level of eHL [13]. The tool has shown robust construct validity and reliability [14]. Cronbach’s alpha was 0.81.

#### The general self-efficacy scale (GSES)

The GSES is a psychological assessment tool measuring the participants' belief in their ability to handle challenges and accomplish goals [15]. Consisting of 10 questions, scores range from 10 to 40, with higher scores indicating higher self-perceived self-efficacy. Scores were normalized to a 1–4 scale in this study. The GSES has shown validity and reliability in studies on patients with different conditions [15]. Cronbach’s alpha was 0.91.

### Statistical analysis

Data analyses were performed using Stata 18.0. General characteristics were summarized using means and standard deviations or median and minimum and maximum values as appropriate. To examine possible relationships between HLQ-p, eHEALS, and various parental and child factors, bivariate correlation (Pearson's R) was utilized. Next, a hierarchical linear multiple regression analysis in four steps was performed using the enter method. Step 1 included child sex and comorbidity; Step 2 included parental sex and age; Step 3 included living arrangements, language, and education; and Step 4 involved adjustment for GSES score. The included variables were based on results from the bivariate correlation. The associations are presented as standardized beta coefficients and adjusted R^2^ explained variation in the associations. Significance was set at *p* < 0.05. The online form ensured no missing data for the HLQ-p, GSES, and study-specific questionnaire by making responses mandatory. Missing data for eHEALS was 7%, which was deemed acceptable for the analysis.

### Ethics

The project was ethically approved by the Regional Committee for Medical Ethics (REK; 402,216) and the Hospital’s Data Protection Officer (22/03367). All parents gave written consent. The children received age-appropriate information about the project.

## Results

### Cohort characteristics

**Fig. 1:**
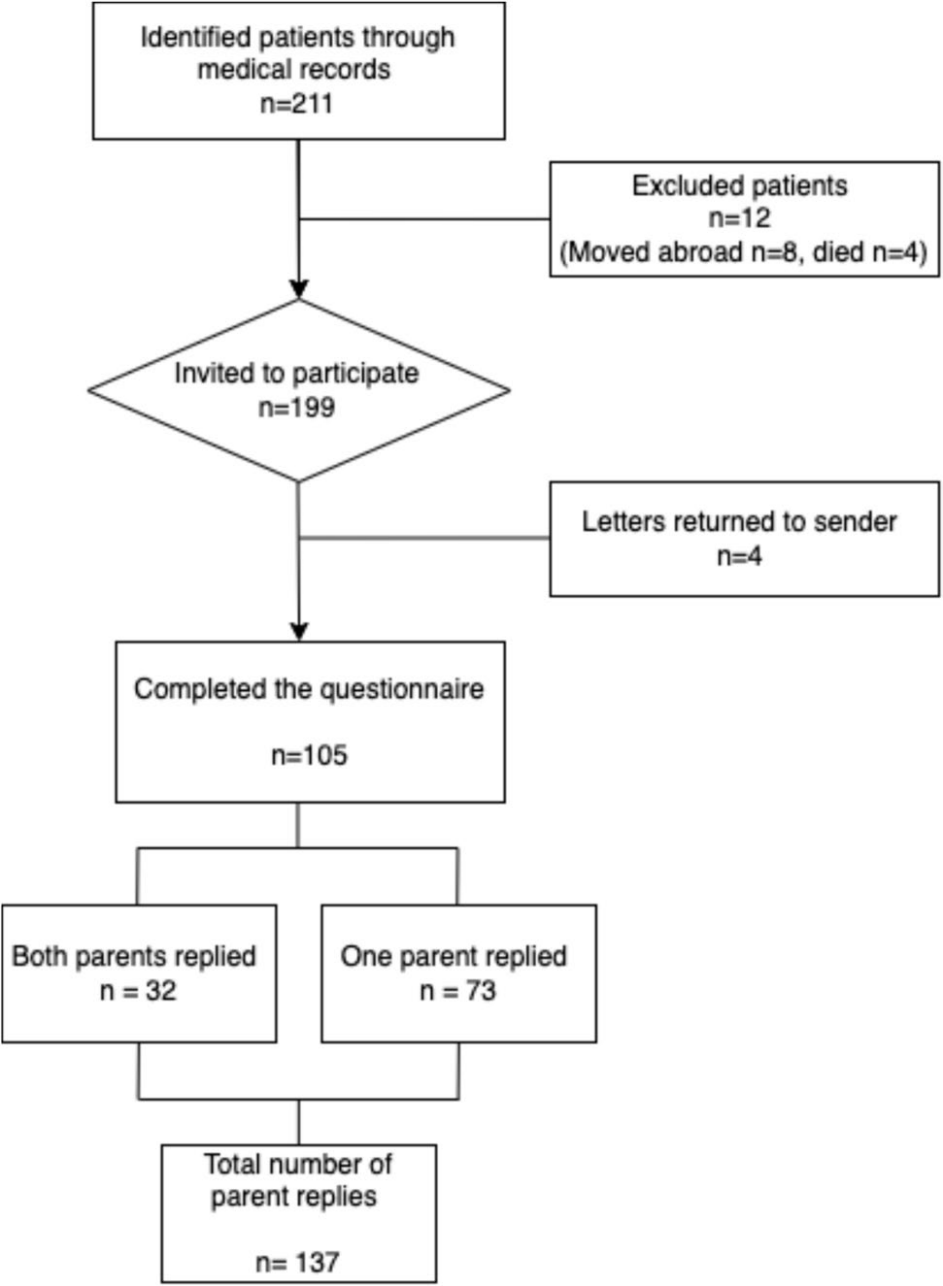
Flowchart of the recruitment process of invited parents of children with anorectal malformation

Parents of 105 out of 199 (53%) children completed the questionnaire. We received 137 parent responses, of which 55 (40%) were from fathers. In 32 patients, both parents participated (Fig. 1).

The median parental age was 38 (21–57) years, with fathers being significantly older than mothers (41 versus 37 years, *p* = 0.02). Most parents lived with the other parent of the child (68%), had higher education (51%), and worked full-time (74%). 34% did not speak Norwegian at home or combined another language with Norwegian at home. The mean age of the children was 7.1 (SD 4.7) years (Table 1).

**Table 1:** Demographic characteristics of 105 children with anorectal malformation, whose parents’ health literacy was examined

Variable	
*Type of ARM*	
Perineal fistula	42 (40.0%)
Vestibular fistula	19 (18.1%)
Urethral fistula	26 (24.8%)
No fistula	11 (10.5%)
Cloacal malformation	5 (4.8%)
Anal stenosis	2 (1.9%)
Male sex (n, %)	50 (57.1%)
Age (mean ± SD)	7.1 (4.7)
VACTERL association (n, %)	53 (50.5%)

*ARM* anorectal malformation. *VACTERL* vertebral, anorectal, cardiac, tracheoesophageal, renal, and limb anomalies

### General knowledge about anorectal malformation

**Fig. 2:**
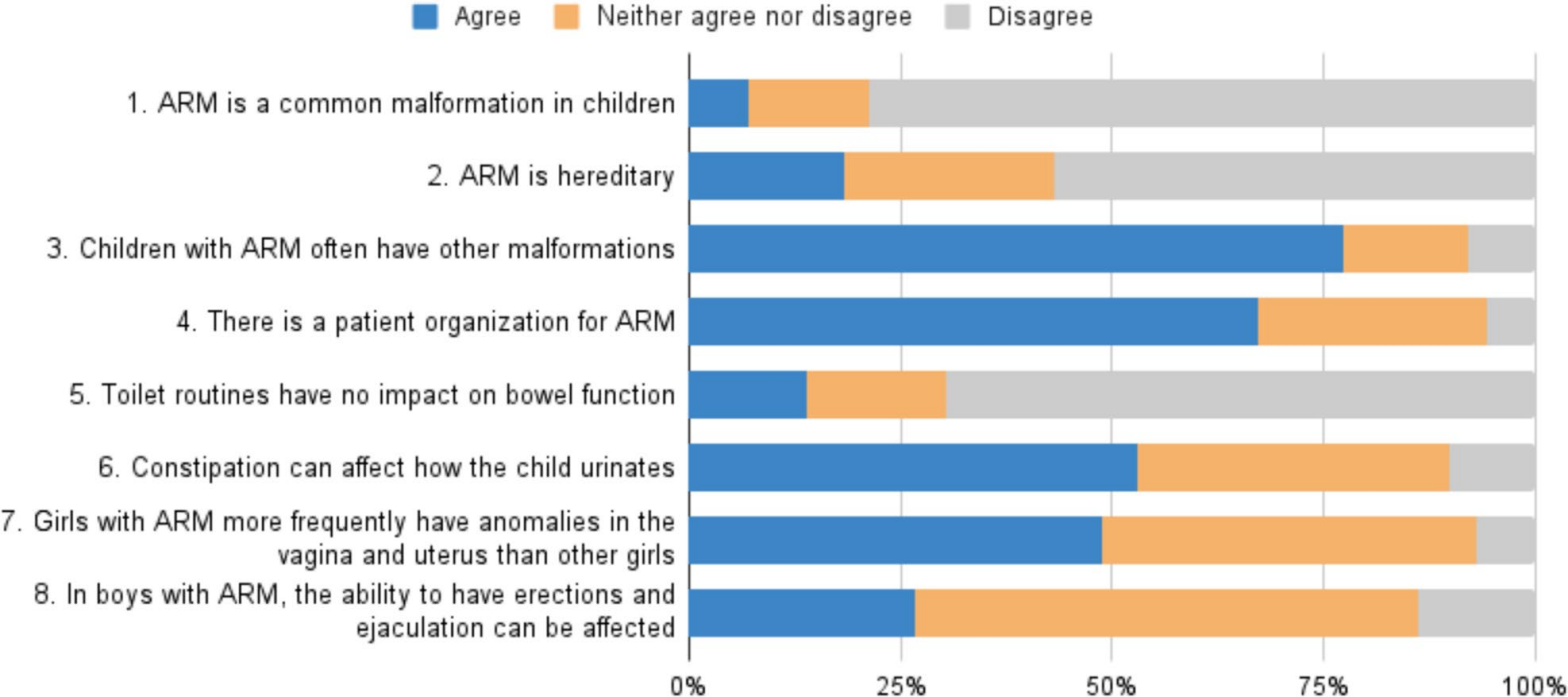
Parents’ general knowledge about anorectal malformations

Generally, the parents showed good disease-specific knowledge about ARM (Fig. 2). For example, 80% of parents acknowledged that ARM is an uncommon malformation, and 70% were aware that many children with ARM have additional anomalies. Furthermore, 90% knew about the existence of the national patient organization for ARM.

### Health literacy, eHEALS and self-efficacy scores

The HLQ-p domains with the highest scores were “actively managing my child’s health” and “active engagement” (domains 3 and 6) (Table 2). The domains with the lowest scores were “having sufficient information”, “social support”, and “appraisal of health information” (domains 2, 4, and 5). HL challenges varied among the nine domains. Only 2% had challenges in active management, whereas 37% of parents had challenges in interpreting health information, 18% in having enough information, and 39% had challenges in social support.

**Table 2:** Descriptive statistics of Health Literacy Questionnaire-Parent (HLQ-p), the e-Health Literacy Scale (eHEALS) and the General Self-Efficacy Scale (GSES) of parents of children with an anorectal malformation

HLQ-p domains	n (%)	Mean (SD)	Cronbach α
1. Feel that providers understand/support my child’s situation	137 (100)	3.0 (0.5)	0.74
2. Having sufficient information to manage my child’s health	137 (100)	2.9 (0.6)	0.72
3. Actively managing my child’s health	137 (100)	3.2 (0.4)	0.75
4. Experience social support for my child’s health	137 (100)	2.6 (0.6)	0.76
5. Appraisal of health information	137 (100)	2.7 (0.5)	0.77
6. Ability to actively engage with healthcare providers	137 (100)	3.6 (0.6)	0.79
7. Navigating the healthcare system	137 (100)	3.4 (0.6)	0.84
8. Ability to find good health information	137 (100)	3.5 (0.6)	0.78
9. Understand health information well enough to know what to do	137 (100)	3.5 (0.7)	0.83
eHEALS, ability to use technology in health	127 (93)	3.7 (0.6)	0.81
GSES, self-efficacy	137 (100)	2.9 (0.5)	0.91

Max score for domains 1–5 was 4, and for domains 6–9, the max score was 5

The parents scored relatively high in eHL (mean 3.7, SD 0.6, max score 5). Thus, most parents knew how to use electronic resources for managing their child’s health. Concerning self-efficacy, the mean GSES score was 2.9 (SD 0.5, max score 4), and 48% had scores below 3, indicating reduced self-efficacy. Mothers and fathers had comparable GSES scored (2.8 vs 2.9, *p* = 0.3).

### Factors influencing health literacy

Bivariate correlation analyses identified that several factors influenced parental HL. Child-related factors negatively impacting HL included having a female child and a child with comorbidities. Factors like type of ARM, time since diagnosis, age at surgery, number of surgeries, and presence of a stoma did not influence HL scores and were excluded from further regression analysis. Parental factors linked to higher HL scores included being female, over 40 years old, having higher education, living with the child's other parent, speaking only Norwegian at home, and having high self-efficacy (Supplementary 1).

Multivariate regression uncovered several predictors for HL (Table 3). Having a female child predicted lower scores in social support, critical appraisal, and understanding health information (domains 4, 5, and 9, St. β 0.2–0.4). Child comorbidity was linked to lower scores in finding and understanding health information (domains 8 and 9, St. β − 0.2). Mothers scored higher in active management, critical appraisal, and understanding health information (domains 3, 5, and 9, St. β 0.2) and had higher overall eHEALS scores. Parents over 40 scored higher in having sufficient information and finding health information (domains 2 and 8, St. β 0.2). Non-Norwegian-speaking parents scored lower in actively managing their child's health (domains 3 and 6, St. β 0.2), and those not living with the co-parent scored lower in social support (domain 4, St. β 0.2). Higher education was associated with better scores in feeling supported by HCP (domains 1, 2, 7, and 9, St. β 0.2–0.4), and high self-efficacy was significantly associated with higher scores across all HLQ-p domains.

**Table 3:** Step 4 in the regression model demonstrating standardized beta coefficients (St. β), significance (*) and *R*^*2*^/adjusted *R*^*2*^ for the Health Literacy Questionnaire-Parent domains and the independent variables

	HPS St. β	HSI St. β	AMH St. β	SS St. β	CA St. β	AE St. β	NHS St. β	FHI St. β	UHI St. β	eHEALS St. β
Female child	− 0.04	− 0.03	− 0.14	− 0.35*	0.25*	− 0.13	− 0.05	− 0.12	− 0.17*	− 0.11
Child comorbidity	− 0.06	0.11	0.03	− 0.72	0.11	0.04	0.03	− 0.20*	− 0.18*	0.03
Parental female sex	0.09	0.14	0.20*	− 0.11	0.16*	0.02	0.09	0.19	0.19*	0.22*
Parental age > 40	0.01	0.23*	0.01	0.05	0.01	0.01	0.01	0.21*	0.04	0.01
Spoke Norwegian at home	− 0.06	0.02	0.15*	0.09	0.01	0.17*	− 0.05	− 0.07	− 0.05	0.12
Living with co-parent	− 0.09	− 0.11	0.01	0.23*	0.07	− 0.16	− 0.12	− 0.10	− 0.06	0.14
High education	0.23*	0.29*	0.13	0.16	0.04	0.17	0.25*	0.11	0.43*	0.19
High self-efficacy	0.25*	0.38*	0.24*	0.47*	0.48*	0.66*	0.58*	0.63*	0.64*	0.34*
R ^2^ , adjusted R^2^	0.2, 0.2	0.4, 0.4	0.2, 0.2	0.4, 0.3	0.4, 0.4	0.5, 0.5	0.5, 0.4	0.5, 0.5	0.6, 0.6	0.2, 0.1

Bold indicates signify negative coefficients

Bolditalic indicates positive significant beta coefficients

*HPS* feel that healthcare providers understand and support my child’s situation, *HSI* having sufficient information to manage my child’s health, *AMH* actively managing my child’s health, SS social support for health, *CA* Appraisal of health information, *AE*: ability to actively engage with healthcare providers, *NHS* navigating the healthcare system, *FHI* ability to find good health information, *UHI* understand health information well enough to know what to do, *eHEALS*: electronic health literacy scale

*Statistically significant *p*-value (*p* < 0.05)

## Discussion

This study is the first to explore parental HL within the context of ARM. HL challenges were primarily found in domains related to information and social support. The parents reported lack of adequate information and struggled to interpret the information they received. The design of this study did not allow us to identify what kind of information the parents missed or found difficult to understand. That the parents demonstrated solid basic knowledge about ARM, suggests that the information they got about the malformation and the surgical treatment probably was adequate. This aligns with studies showing that parents of children undergoing surgery, generally, are satisfied with the information about surgical details [16, 17]. Therefore, it is likely that parents are missing information about factors not directly related to the anatomical malformation or the surgical treatment. This information may include the impact on QoL, strategies for managing the condition at school, ways to support their child’s emotional well-being, or available resources for assistance. Like parents of ARM children, parents of children with Hirschsprung disease and epilepsy also reported lack of information and difficulties in interpreting information when HL was examined [18, 19]. All these parents have children with chronic diseases that may influence daily life for the child and the family, and it is possible that information about how to handle this is given too little attention.

There is growing recognition that children with chronic diseases and their families need more than medical treatment to handle everyday life. Therefore, multidisciplinary follow-up for congenital conditions is recommended [20]. In Norway, patients with ARM and their families meet a multidisciplinary team at the hospital, there is an active and well-known patient organization, as well as a center for rare diagnoses that includes ARM in its portfolio. Even so, the parents of ARM children scored lower in the information domains than parents of children with Hirschsprung disease [18]. This finding was somewhat surprising to us, since Hirschsprung disease has only recently been added to the center for rare diseases’ portfolio, and the patient organization is new and less known among families. Although patients with ARM and Hirschsprung face many similar challenges, ARM patients generally have more comorbidities resulting in a more complex information load from HCP. Additionally, the complexity of the malformation itself may be difficult to handle. Nevertheless, the results indicate that communication with parents needs improvement, and further studies are necessary to clarify the specific information that parents want.

The parents scored low in the domain of social support. The perceived lack of social support among parents of ARM patients is well documented in studies from different countries [5, 6, 21]. The rarity of the condition and the stigma around bowel problems are plausible explanations [22, 23]. Challenges concerning social support were also addressed by parents of children with Hirschsprung disease which have many similar bowel problems as those with ARM [18, 24].

### Predictors of health literacy

This study revealed that several factors were associated with parental HL levels. Of child-related factors, having a daughter or a child with comorbidities seemed to be most important. Having a daughter with ARM predicted lower parental HL. This finding aligns with previous research consistently showing that girls with ARM and other colorectal conditions experience more psychosocial and mental health problems compared to boys [24–26]. Furthermore, girls with ARM report lower self-efficacy and disease-specific functioning, both of which can impact the quality of life [27]. The reasons for these gender-based differences and their connection to parental HL is unclear. One possible explanation is that parents of children with mental health and psychosocial problems feel less successful in their parenting roles, thereby undermining their self-efficacy and HL, or vice versa [28, 29]. There is a need for larger studies that explore the relationship between gender and parental HL. Having a child with comorbidities was also associated with lower HL in the domains related to finding and understanding health information. This is not unexpected, as similar findings have been found for parents of children with Hirschsprung disease and complex epilepsy [12, 19]. Having a child with comorbidity may cause feelings of being overwhelmed and exhausted, resulting in a poorer ability to filter and process complex medical information. Furthermore, the multitude of medical information available online and through social media may exacerbate this [30].

Several parental factors influenced HL, with low socioeconomic status, particularly lower education level being the most significant determinant. This aligns with findings in much of the existing literature [11]. Lower education level may correlate with other social determinants of health, such as income, living conditions, and healthcare access [32]. However, higher education does not ensure higher HL, as many parents in this study with higher education also reported HL problems. When looking at gender, mothers outperformed fathers in areas of active management, critical appraisal, and understanding health information. This trend aligns with existing research suggesting that fathers have more HL challenges than mothers [19, 33]. One study found that fathers had higher communicative HL than mothers; however, they were also more educated [38]. Not living with the co-parent predicted lower HL in the domain of social support. This is not surprising as non-cohabitating parents may have fewer recourses and encounter more communication barriers. Previous research supports our findings as having unmarried parents has been linked to failed bowel management for children with ARM and poorer outcome in children with Hirschsprung disease [34, 35].

Nearly one-fifth of Norway’s population is first- or second-generation immigrants, a demographic known to face significant HL challenges [36]. Our findings support this, revealing that parents who did not speak Norwegian at home, or used it alongside another language, generally tended to have lower HL. This group displayed HL challenges particularly in their ability to actively engage with HCP and effectively managing their child's health. Cultural and even ethnical differences between patients and providers can contribute to misunderstandings, opposing health conceptions, and poorer postoperative outcomes [37, 38]. Notably, our study likely underestimates these challenges, as parents unable to complete the questionnaires in Norwegian were excluded.

Self-efficacy emerged as a strong predictor of parental HL. Similar findings have been reported in other pediatric patient groups [11, 19]. Self-efficacy, a person’s belief in their ability to complete a task or achieve a goal, plays a crucial role in HL. It empowers individuals to understand and apply information effectively, facilitating better health decisions and outcomes [29]. Unlike fixed sociodemographic factors such as education level, self-efficacy is a dynamic trait that can be responsive to intervention.

Parents exhibited high levels of eHL. High levels of eHL have also been reported by parents of children with other surgical conditions [18, 40]. Families of children with ARM and Hirschsprung disease using eHealth established earlier and more frequent contact with HCP, enabling more tailored treatment and increased parental involvement [41].

### Strengths and weaknesses

A key strength of this study is its diverse representation of the parent population and the inclusion of a high number of fathers and non-native speakers. It is also a strength that both online and paper questionnaires were offered. The weaknesses of the study include the relatively small sample size, the absence of data on non-responders, and no information on HL of non-Norwegian-speaking parents. Furthermore, the limitation of the cross-sectional design is that it cannot establish causality. Another limitation of the study is the small number of paired parental responses, which prevented us from comparing mothers’ and fathers’ perspectives on the same child.

## Conclusion and implications

This study shows that parents of children with ARM have HL challenges, particularly regarding information access, interpretation, and social support. Further research is necessary to determine what specific information these parents are missing and how it should be presented for better understanding. We have identified characteristics associated with low HL, highlighting the need for increased attention to young parents, those with low education, multilingual parents, and single or divorced parents. Additionally, having a daughter or a child with comorbidities seems to correlate with greater HL challenges. Based on these findings, institutions should focus on identifying parents in at-risk groups and provide them with targeted support. There should also be a low threshold for using interpreters when necessary. Furthermore, improving self-efficacy may potentially enhance parental HL, and digital tools may be helpful in enhancing HL.

## Supplementary Information

Below is the link to the electronic supplementary material.

Supplementary file1 (DOCX 18 KB)

## Data Availability

No datasets were generated or analysed during the current study.
