# Supplementary material for: Parental health literacy in anorectal malformation: needs and challenges
Source: Pediatr Surg Int. 2025 Jul 16;41(1):214. doi: 10.1007/s00383-025-06096-6 (PMC12267356; doi:10.1007/s00383-025-06096-6)
Supplement: Supplementary file 1 — Supplementary file1 (DOCX 18 KB) [file 383_2025_6096_MOESM1_ESM.docx]

**Supplementary 1**

Bivariate correlation coefficients (Pearson’s R) and significance levels (*) between the Health Literacy Questionnaire-Parent (HLQ-p) domains, the electronic health literacy scale (eHEALS), and parental factors were analyzed. A significance level of p<0.05 was applied.

|  | Female child | Child  Comor-bidity | Being female | Age >40 | Only Norwegian at home | Living with co- parent | High education | High self-efficacy |
| --- | --- | --- | --- | --- | --- | --- | --- | --- |
| HLQ-p domains | *r* | *r* | *r* | *r* | *r* | *r* | *r* | *r* |
| 1. Feel that healthcare providers understand and support my child's health | -0 | -0.1 | 0.1 | -0.1 | -0.2 | -0.2 | -0.3* | -0.3* |
| 2. Having sufficient information to manage my child's health | -0 | -0.2* | -0.1 | -0.1 | -0 | -0.2 | -0.4* | -0.4** |
| 3. Actively managing my child's health | 0 | 0 | 0.2* | 0.2 | 01 | 0 | 0.2* | 0.3* |
| 4. Experience social support for my child's health | 0.2* | 0 | 0.1 | 0 | 0.2 | 0.3* | 0.3* | 0.3* |
| 5. Appraisal for health information | 0.2* | 0.2* | 0.1 | 0.1 | 0 | -0.1 | -0.1 | 0.4** |
| 6. Ability to actively engage with healthcare providers | -0.1 | 0 | 0 | 0.2* | 0.2* | 0.3* | 0.3* | 0.5** |
| 7. Navigating the healthcare system | -0.1 | 0.1 | 0.1 | 0.1 | 0.1* | 0.2* | 0.4* | 0.5** |
| 8. Ability to find good health information | -0.1 | 0.3 | 0.2 | 0.2 | 0.2 | 0.2 | 0.2* | 0.6** |
| 9. Understand health information well enough to know what to do | -0.2 | 0.3* | 0.2* | 0.3* | -0.1 | 0.1 | 0.5** | 0.6** |
| eHEALS | -0.1 | 0 | 0.2* | 0.2* | 0.1 | 0.2 | 0.2* | 0.4* |

*Significant at >0.01 level, **Significant at >0.001 level, r = Bivariate Correlations (Pearson’s R)
HCP: healthcare providers
